# Defining the Antimalarial Activity of Cipargamin in Healthy Volunteers Experimentally Infected with Blood-Stage Plasmodium falciparum

**DOI:** 10.1128/AAC.01423-20

**Published:** 2021-01-20

**Authors:** James S. McCarthy, Azrin N. Abd-Rahman, Katharine A. Collins, Louise Marquart, Paul Griffin, Anne Kümmel, Aline Fuchs, Cornelis Winnips, Vishal Mishra, Katalin Csermak-Renner, J. Prakash Jain, Preetam Gandhi

**Affiliations:** a QIMR Berghofer Medical Research Institute, Herston, Queensland, Australia; b Q-Pharm Pty. Ltd., Herston, Queensland, Australia; c Department of Medicine and Infectious Diseases, Mater Hospital and Mater Research, South Brisbane, Queensland, Australia; d The University of Queensland, Brisbane, Queensland, Australia; e IntiQuan GmbH, Basel, Switzerland; f Medicines for Malaria Venture, Geneva, Switzerland; g Novartis Pharma AG, Basel, Switzerland; h Novartis Institutes for Biomedical Research, Emeryville, California, USA; i Novartis Healthcare Pvt. Ltd., Hyderabad, India

**Keywords:** *Plasmodium falciparum*, antimalarial agents, malaria

## Abstract

The spiroindolone cipargamin, a new antimalarial compound that inhibits *Plasmodium* ATP4, is currently in clinical development. This study aimed to characterize the antimalarial activity of cipargamin in healthy volunteers experimentally infected with blood-stage Plasmodium falciparum. Eight subjects were intravenously inoculated with parasite-infected erythrocytes and received a single oral dose of 10 mg cipargamin 7 days later. Blood samples were collected to monitor the development and clearance of parasitemia and plasma cipargamin concentrations. Parasite regrowth was treated with piperaquine monotherapy to clear asexual parasites, while allowing gametocyte transmissibility to mosquitoes to be investigated. An initial rapid decrease in parasitemia occurred in all participants following cipargamin dosing, with a parasite clearance half-life of 3.99 h. As anticipated from the dose selected, parasite regrowth occurred in all 8 subjects 3 to 8 days after dosing and allowed the pharmacokinetic/pharmacodynamic relationship to be determined. Based on the limited data from the single subtherapeutic dose cohort, a MIC of 11.6 ng/ml and minimum parasiticidal concentration that achieves 90% of maximum effect of 23.5 ng/ml were estimated, and a single 95-mg dose (95% confidence interval [CI], 50 to 270) was predicted to clear 10^9^ parasites/ml. Low gametocyte densities were detected in all subjects following piperaquine treatment, which did not transmit to mosquitoes. Serious adverse liver function changes were observed in three subjects, which led to premature study termination. The antimalarial activity characterized in this study supports the further clinical development of cipargamin as a new treatment for P. falciparum malaria, although the hepatic safety profile of the compound warrants further evaluation. (This study has been registered at ClinicalTrials.gov under identifier NCT02543086.)

## Abstract (precis)

The spiroindolone cipargamin, a new antimalarial compound that inhibits *Plasmodium* ATP4, is currently in clinical development. This study aimed to characterize the antimalarial activity of cipargamin in healthy volunteers experimentally infected with blood-stage Plasmodium falciparum.

## INTRODUCTION

Despite efforts at progress toward eradicating malaria, it is still responsible for a large burden of morbidity and mortality worldwide (1). Achieving the long-term goal of malaria elimination is hampered by many factors, particularly the development and spread of antimalarial drug resistance (2). The development of new antimalarial treatments with novel mechanisms of action is a high priority, with several candidates currently under clinical development (3). The ideal antimalarial treatment would be delivered as a single dose (to avoid compliance issues associated with multidose treatment regimens), have activity against multiple parasite life cycle stages (including the transmissible sexual stage), and prevent new infection.

The spiroindolone compound cipargamin (formerly KAE609 or NITD609) was identified by high-throughput screening (4) and represents the first of a new class of antimalarial compounds. Spiroindolones are thought to exert their antiplasmodial activity through inhibition of the cation ATPase PfATP4, which leads to disruption of parasite sodium homeostasis (5). *In vitro* studies indicated that cipargamin is active against both asexual and sexual stages of Plasmodium falciparum (6), and single-dose cure was demonstrated in a rodent malaria model (7). The safety, tolerability, and pharmacokinetics (PK) of cipargamin in healthy adults were assessed using a single- and multiple-ascending-dose study design (8). The compound was found to be tolerated in humans when administered as a single dose up to 300 mg or up to 150 mg daily for 3 days, although transient gastrointestinal and genitourinary adverse events (AEs) were observed with increasing frequency at higher doses. The human pharmacokinetic data, together with data from a preclinical pharmacokinetic/pharmacodynamic (PK/PD) analysis using the Plasmodium berghei malaria mouse model (9), predicted that a 30-mg daily dose may be sufficient to clear parasitemia in malaria patients. Indeed, in a subsequent phase 2 study in Thailand, the efficacy of a 3-day regimen of 30 mg cipargamin daily was demonstrated to clear parasitemia from patients infected with P. falciparum or Plasmodium vivax (10).

The ability to investigate the activity of new antimalarial candidate compounds in healthy volunteers is a useful tool in the antimalarial drug development toolkit. Volunteer infection studies (VIS) using the induced blood-stage malaria (IBSM) model, where healthy adults are experimentally inoculated with blood-stage malaria parasites, allow for early characterization of a compound’s pharmacological activity and tolerability in a controlled setting. Furthermore, determination of a compound’s PK/PD relationship using VIS enables informed dose selection for efficacy trials in malaria patients, potentially reducing overall development timelines. VIS have been used successfully during the clinical development of several new antimalarial drug candidates (11–17).

The primary objective of the current study was to characterize the antimalarial activity of cipargamin in a VIS using the P. falciparum IBSM model in order to aid in optimizing future therapeutic dosing. A key component of this was to define the PK/PD relationship between cipargamin PK and parasite clearance in healthy volunteers. Additional objectives were to evaluate the safety/tolerability of cipargamin and investigate the ability of cipargamin to interrupt transmission of P. falciparum to mosquitoes.

## RESULTS

### Subject disposition.

This study was conducted between July and September 2015. A single cohort of eight healthy subjects was inoculated with P. falciparum-infected erythrocytes on day 0. All subjects were male, and the mean age was 27.9 years (Table 1). All subjects received a single 10-mg dose of cipargamin on day 7, were subsequently dosed with 960 mg piperaquine in response to first parasite regrowth, and received standard antimalarial treatment with artemether/lumefantrine and primaquine prior to the end of the study.

**TABLE 1:** Demographic profile of the 8 subjects in this study

Characteristic	Value
Age, mean yr (SD)	27.9 (11.8)
Sex, *n* males (%)	8 (100.0)
Race, *n* (%)	
White	4 (50.0)
Other	4 (50.0)
Ethnicity, *n* (%)	
Hispanic or Latino	2 (25.0)
Other	6 (75.0)
Body mass index, mean kg/m^2^ (SD)	25.1 (2.7)
Ht, mean cm (SD)	177.8 (3.9)
Wt, mean kg (SD)	79.4 (8.5)

### Safety.

A total of 89 adverse events (AEs) were reported, with all 8 subjects experiencing at least one AE (Table 2). The most common AEs were signs and symptoms frequently associated with malaria, including pyrexia (6 subjects), myalgia (5 subjects), and headache (5 subjects) (see Table S1 in the supplemental material). The majority of AEs were mild in severity (68/89 [76.4%]); there were 18 moderate AEs and 3 severe AEs. The severe AEs were 2 cases of abnormal liver function tests (LFTs) in 2 subjects (considered potentially related to cipargamin), and one case of anorexia (not considered related to cipargamin).

**TABLE 2:** Summary of adverse events for the 8 subjects in this study

Type of AE^*a*^	No. (%) of subjects with at least 1 AE	No. of AEs
Any AEs	8 (100)	89
Cipargamin related	4 (50.0)	10

Moderate AEs (grade 2)	7 (87.5)	18
Cipargamin related	2 (25.0)	2

Severe AEs (grade 3)	2 (25.0)	3
Cipargamin related	2 (25.0)	2

AEs leading to discontinuation	0 (0)	0

SAEs	3 (37.5)	3

a AE, adverse event; SAE, serious adverse event. The severity of AEs was graded in accordance with the *Common Terminology Criteria for Adverse Events*, version 4.03.

There were 3 serious adverse events (SAEs) reported in the study, which included the 2 cases of severe LFT abnormalities mentioned above. The third SAE was another case of abnormal LFT results (considered potentially related to cipargamin), which was moderate in severity. Although a relationship between the SAEs and cipargamin was suspected, other administered drugs (paracetamol and piperaquine) and the induced malaria infection in previously malaria-naive individuals were also considered to be possible contributory factors. These serious LFT abnormalities are described in further detail below.

There were no clinically significant abnormalities in biochemistry parameters other than the LFT findings mentioned above. Additionally, no clinically significant abnormalities were observed in hematology, urinalysis, or electrocardiogram (ECG) parameters. The severity of malaria symptoms and signs experienced by participants during the study assessed by the malaria clinical scoring tool are presented in Table S2 in the supplemental material. At the time of cipargamin dosing, subjects exhibited no or negligible malaria symptoms or signs (clinical score of 0 or 1). Six subjects developed mild to moderate symptoms or signs of malaria around the time of piperaquine administration in response to parasite regrowth (3 to 9 days after cipargamin dosing; clinical scores of 3 to 13).

### Liver function test abnormalities.

The subject with the most severe LFT abnormalities was a 52-year-old subject (subject 3; see Fig. S1 in the supplemental material) who developed elevations in LFTs (which partially met the criteria for a potential Hy’s law case) 8 days after dosing with cipargamin, with a raised bilirubin level at 74 μmol/liter (3.7 times the upper limit of normal [3.7 × ULN]), conjugated bilirubin at 22 μmol/liter (3.1 × ULN), aspartate transaminase (AST) at 106 U/liter (2.6 × ULN), alanine transaminase (ALT) at 187 U/liter (4.6 × ULN), and gamma-glutamyl transferase (GGT) at 215 U/liter (4.3 × ULN). The subject received paracetamol as symptomatic treatment for malaria. Physical examination was notable for transient mild tender liver (duration of 3 days), which began 5 days after the onset of the LFT abnormality. The subject otherwise remained asymptomatic, with no clinical features of acute hepatitis (anorexia or nausea), or other evidence of decompensation (abnormal coagulation profile). His liver tests completely normalized over a period of approximately 6 weeks. Ultrasonography showed fatty liver but ruled out choledocholithiasis, a genetic test for Gilbert’s syndrome was negative, and a liver FibroScan did not indicate evidence of advanced fibrosis or cirrhosis. Careful history taking by an external consultant hepatologist revealed a previous and undisclosed history of excessive alcohol intake. As the total bilirubin mostly comprised unconjugated bilirubin, the LFT elevations were inconsistent with drug-induced liver injury, being more likely explained by hemolysis or impaired bilirubin conjugation.

The second case of severe abnormality in LFTs occurred in a 22-year-old subject (subject 8; Fig. S1) 11 days after dosing with cipargamin. A concomitant elevation in ALT, AST, and GGT was observed, with a peak ALT value of 964 U/liter (24.1 × ULN), a peak AST value of 443 U/liter (11 × ULN), and a peak GGT value of 159 U/liter (3.1 × ULN). No elevation in bilirubin was observed. The subject received paracetamol for malaria symptoms, and the elevated liver function tests normalized over the course of approximately 2 weeks. Tests for hepatitis, cytomegalovirus, and Epstein-Barr virus were negative, and liver and abdominal ultrasounds were normal. Furthermore, analysis of the subject’s medical history did not reveal any conditions or medication use that may have contributed to the abnormal liver function tests.

A third case of moderate abnormality in LFTs occurred in an 18-year-old subject (subject 1; Fig. S1) 8 days after dosing with cipargamin. Elevated ALT was observed (maximum of 208 U/liter [5.2 × ULN]), along with a mild elevation in AST (maximum of 88 U/liter [2.2 × ULN]); no elevation in bilirubin was observed. The subject remained asymptomatic, and the ALT normalized over the course of approximately 2 weeks without treatment. Analysis of the subject’s medical history did not reveal any conditions or medication use that may have contributed to the ALT elevation.

Together, these three cases of elevated LFT levels resulted in the study being terminated by the sponsor.

### Pharmacokinetics.

**FIG 1:**
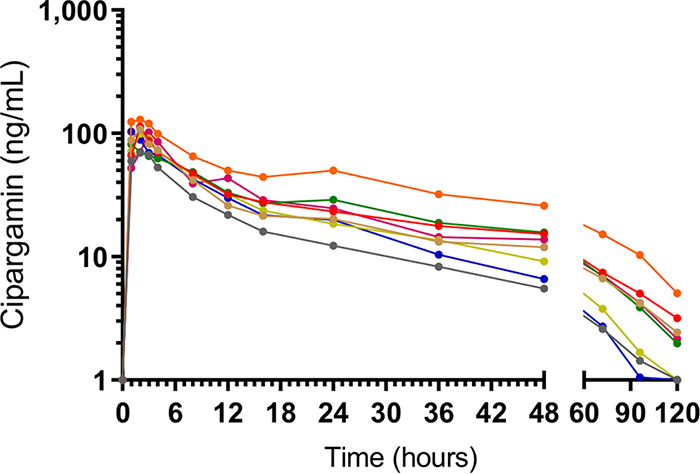
Individual subject cipargamin plasma concentration-time profiles. For the purpose of graphing on a logarithmic scale, time points at which cipargamin could not be detected were assigned a value of 1 ng/ml.

The intersubject variability in plasma cipargamin concentration-time profiles was low to moderate, with the coefficient of variation (CV) less than 40% for all PK parameters (Fig. 1 and Table 3). Absorption was rapid, with the peak plasma concentration occurring 2 h after dosing; the elimination half-life was approximately 26 h.

**TABLE 3:** Plasma pharmacokinetic parameters (noncompartmental analysis) of cipargamin following a single oral dose of 10 mg in the 8 subjects in this study

PK parameter^*a*^	Value
*C*_max_, mean ± SD ng/ml (% CV)	101 ± 18.3 (18.2)
*t*_max_, median h (range)	2.00 (1.00–3.00)
AUC_0–24 h_, mean ± SD h · μg/ml (% CV)	1.01 ± 0.242 (23.9)
AUC_0–∞_, mean ± SD h · μg/ml (% CV)	1.95 ± 0.753 (38.6)
AUC_0–last_, mean ± SD h · μg/ml (% CV)	1.85 ± 0.690 (37.3)
*t*_1/2_, mean ± SD h (% CV)	26.7 ± 6.81 (25.5)
CL/F, mean ± SD liters/h (% CV)	5.72 ± 1.93 (33.7)
*V_z_*/F, mean ± SD liters (% CV)	209 ± 54.5 (26.0)

a CV, coefficient of variation; AUC_0–24 h_, area under the curve up to 24 h; AUC_0–last_, area under the curve up to last time point measure; AUC_0–∞_, area under the curve extrapolated to infinity; *t*_1/2_, elimination half-life; *C*_max_, maximum concentration; *t*_max_, time until *C*_max_ is reached; CL/F, apparent total clearance; *V_z_*/F, apparent volume of distribution during terminal phase.

### Pharmacodynamic response: clearance of P. falciparum parasitemia.

**FIG 2:**
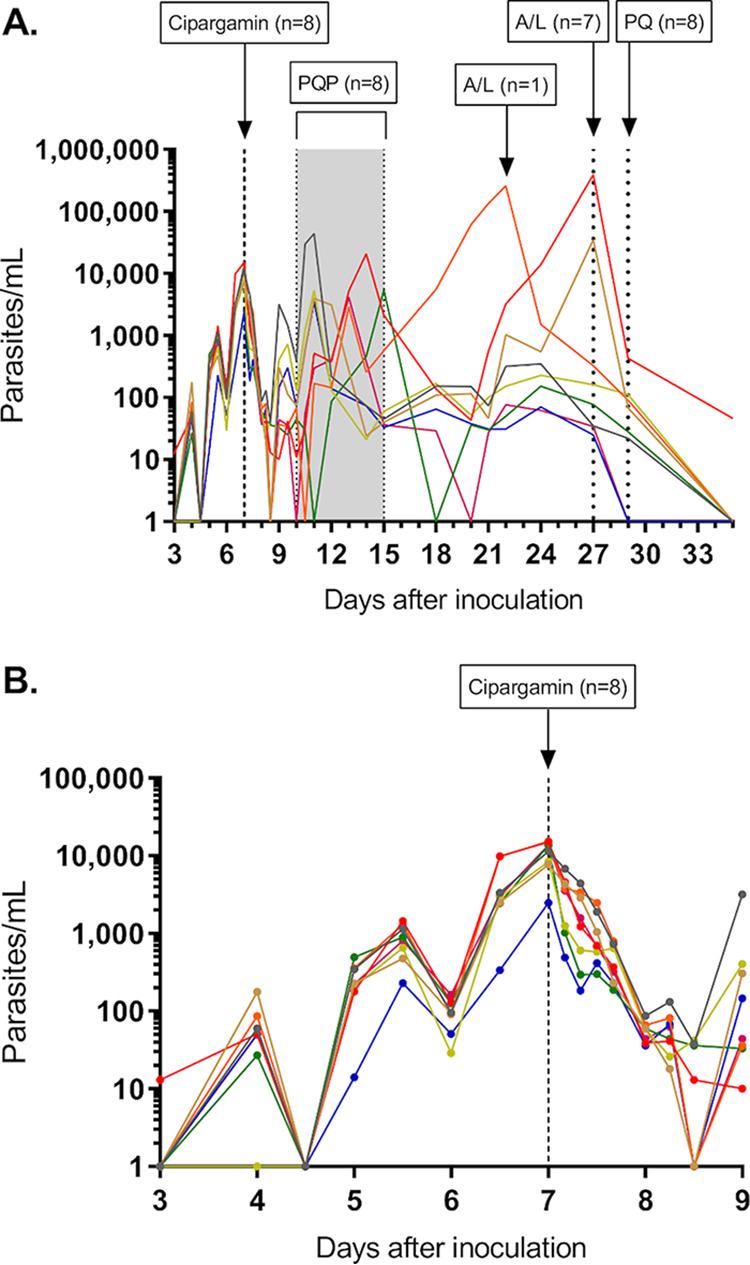
Individual subject parasitemia-time profiles over the entire study (A) and up to 2 days post-cipargamin treatment (B). Subjects were inoculated with ∼1,800 viable parasites, and a single 10-mg dose of cipargamin was administered 7 days later (indicated by the vertical dashed line). A single 960-mg dose of piperaquine (PQP) was administered in response to first parasite regrowth (indicated by the shaded area). Standard therapy with artemether-lumefantrine (A/L) was initiated in response to second parasite regrowth or 20 days after cipargamin dosing if second parasite regrowth did not occur. A single dose of 45 mg primaquine (PQ) was administered prior to the end of study to clear gametocytemia. For the purpose of graphing on a logarithmic scale, time points at which parasitemia could not be detected by qPCR were assigned a value of 1 parasite/ml.

The geometric mean parasitemia (measured using quantitative PCR [qPCR]) upon dosing with 10 mg cipargamin was 9,687 parasites/ml (range, 2,475 to 15,270). A reduction in parasitemia was observed within 24 h of cipargamin administration (Fig. 2A and B). The overall cohort-specific log_10_ parasite reduction ratio over a 48-h time period postdose (PRR_48_) was 3.63 (95% confidence interval [CI], 3.43 to 3.82), which corresponded to a parasite clearance half-life of 3.99 h (95% CI, 3.79 to 4.21). The individual subject log_10_ PRR_48_ and clearance half-life are presented in Table S3 in the supplemental material. Parasite regrowth occurred relatively rapidly in all subjects; piperaquine was administered to all subjects between 3 and 8 days after cipargamin dosing to clear asexual parasites (Fig. 2A).

### Pharmacokinetic/pharmacodynamic analysis.

A two-compartment disposition model with first-order absorption and elimination and no lag time was developed to describe the pharmacokinetics of cipargamin (see Table S4 in the supplemental material). A maximum killing effect (*E*_max_) model best characterized the effect of cipargamin on parasitemia (see Table S5 in the supplemental material). Visual predictive checks of the PK model (see Fig. S2 in the supplemental material) and PK/PD model (see Fig. S3 in the supplemental material) confirmed the adequacy of the models in characterizing the observed data. The efficacy parameters associated with administration of 10 mg cipargamin are presented in Table S6 in the supplemental material. The PK/PD model estimated the median MIC of cipargamin to be 11.6 ng/ml (95% CI, 9.9 to 13.3) and the median minimum parasiticidal concentration that achieves 90% of the maximum effect (MPC_90_) to be 23.5 ng/ml (95% CI, 19.9 to 23.5). Simulations of various single doses of cipargamin (10 to 10,000 mg) in patients with malaria (see Fig. S4 in the supplemental material) resulted in an estimate of 95 mg (95% CI, 50 to 270) as the minimum effective dose needed to clear 10^9^ parasites/ml.

### Gametocytemia and transmission to mosquitoes.

**FIG 3:**
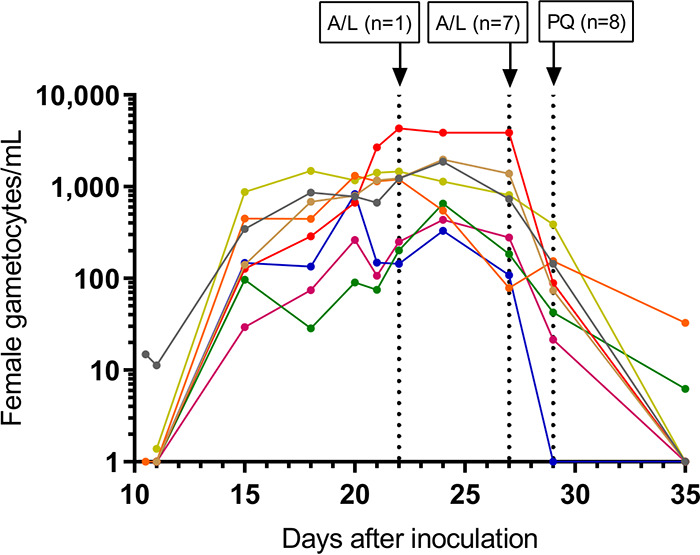
Individual subject gametocytemia-time profiles. Gametocyte density was quantified using qRT-PCR assays specific for female gametocytes (*pfs25*). The vertical dashed lines indicate timing of rescue treatment with artemether-lumefantrine (A/L) and primaquine (PQ). For the purpose of graphing on a logarithmic scale, time points at which gametocytemia could not be detected were assigned a value of 1 gametocyte/ml.

Gametocytes were detected in all 8 subjects following piperaquine administration (Fig. 3). Peak gametocytemia occurred approximately 17 days after cipargamin dosing (24 days postinoculation) for all subjects (geometric mean of 976 female gametocytes/ml; range, 329 to 3,869). To evaluate the transmissibility of gametocytes to mosquitoes, direct membrane feeding assays (DMFAs) were performed 20 days after cipargamin dosing (geometric mean gametocytemia of 439 gametocytes/ml; range, 78 to 3,865). Oocysts could not be detected by qPCR in any mosquito midgut sample, despite the mosquitoes feeding on blood samples successfully. (The mosquito feeding rate was 93.8%, and adult mosquito mortality was 28.2%.) Artemether-lumefantrine and primaquine were administered to all subjects prior to the end of study to clear parasitemia and gametocytemia (Fig. 2A and Fig. 3).

## DISCUSSION

The primary objective of this study was to characterize the antimalarial activity of cipargamin when administered to healthy subjects experimentally infected with blood-stage P. falciparum. A robust assessment of this objective was inhibited by the unexpected safety results observed, which led to premature study termination. It was planned that several doses of cipargamin would be tested and, additionally, that the gametocytocidal activity of cipargamin would be assessed by administering the compound following piperaquine treatment to clear asexual parasites. That a single-dose cohort was enrolled and that the gametocytocidal activity of cipargamin was not assessed are obvious limitations of this study.

The serious abnormalities in LFTs observed in three of the eight subjects who received a single 10-mg dose of cipargamin were unexpected based on preclinical toxicology results (7) and the clinical safety results obtained previously. In the first-in-human study, in which cipargamin was administered to 72 healthy subjects as single doses up to 300 mg or up to 150 mg daily for 3 days, only one subject displayed mild LFT abnormalities (8). This subject received 100 mg of cipargamin once daily for 3 days and developed mild elevations in ALT, AST, lactate dehydrogenase, and creatine kinase, which resolved within 4 days. The subject’s bilirubin level remained within the normal range throughout, and the liver function test abnormalities were not attributed to cipargamin.

Likewise, safety results from two phase 2 studies in which cipargamin was administered to malaria patients did not suggest a major signal associated with LFTs. When cipargamin was administered at a dose of 30 mg per day for 3 days to 11 patients with P. falciparum malaria and 10 patients with P. vivax malaria, three subjects displayed LFT abnormalities (10). Increased levels of ALT and AST occurred in two patients with P. vivax malaria, which were attributed to cipargamin. Peak ALT and AST values were 241 and 106 U/liter on day 9 in one patient and 78 and 63 U/liter on day 6 in the other patient. Additionally, one patient with P. falciparum malaria had increased ALT and AST (192 and 121 U/liter on day 6), although these elevations were not considered to be related to cipargamin because the values were already slightly elevated at screening (54 and 79 U/liter, respectively). No clinically relevant increases in bilirubin level were observed in these patients. When cipargamin was administered to a total of 25 patients with P. falciparum malaria as single doses of 10, 15, 20, or 30 mg, only one patient displayed increased ALT, although 40% of patients displayed increased blood alkaline phosphatase and 20% of subjects displayed hyperbilirubinemia (18).

A third phase 2 study (currently unpublished) in Thai and Vietnamese patients with P. falciparum malaria was designed to assess efficacy and safety of single-dose treatment with cipargamin (75 to 300 mg) and to correlate the outcome with pharmacokinetics (ClinicalTrials.gov identifier NCT01860989). In the first cohort of 11 patients treated with a single 75-mg dose, four patients had asymptomatic transient ALT elevations (>3 × ULN). The sponsor terminated the study after the first dose cohort and initiated a dedicated dose escalation safety study to explore the hepatic profile of cipargamin in patients with acute uncomplicated P. falciparum malaria (ClinicalTrials.gov identifier NCT03334747). This study has recently been completed, and the results will be published separately.

Attributing the causality of LFT abnormalities in clinical trials involving administration of an experimental drug to individuals with malaria is difficult given that such abnormalities are known to be a consequence of the inflammatory process associated with both experimental malaria infection as well as natural infection. In a comprehensive review of LFT results in experimental malaria infection studies where the infection was induced by sporozoite or by blood-stage challenge, abnormalities were observed by both means of infection, and with a variety of approved and experimental antimalarials (19). Across the IBSM studies, ALT was elevated (>2.5 × ULN) in 17.5% of subjects (20/114) and was observed in all eight studies using seven different experimental antimalarial compounds and with piperaquine alone. Furthermore, ALT elevations of >10 × ULN occurred in 5.2% of subjects (6/114) in four studies using different experimental antimalarial compounds. AST was elevated (>2.5 × ULN) in 11.4% of subjects (13/114) in six of the eight IBSM studies. Likewise, a retrospective review of LFT results among mostly malaria-naive travelers returning to Queensland, Australia, with P. falciparum or P. vivax infection indicated that LFT changes are not uncommon if testing is done in the 7- to 10-day window after treatment (20). Finally, a recent review of LFT results in P. vivax IBSM studies observed that LFT changes were more common after treatment when there was a rapid clearance of parasite biomass (28).

Despite the small sample size and the fact that only a single dose level was examined in this study, valuable information was obtained on the antimalarial activity of cipargamin. A single 10-mg dose resulted in a rapid initial reduction in parasitemia in all 8 subjects. The cohort-specific parasite clearance half-life of 3.99 h was faster than the parasite clearance half-lives of several candidate and marketed antimalarial drugs administered as single doses in IBSM studies: 5.22 h for 640 mg piperaquine (17), 6.2 h for 10 mg/kg mefloquine (13), 6.5 h for 200 mg artefenomel (12), 6.5 h for 800 mg ferroquine (14), and 9.4 h for 150 mg DSM265 (13). The parasite clearance half-life following administration of 10 mg cipargamin in the current IBSM study (3.99 h) was similar to that observed when the equivalent dose was administered to patients with P. falciparum malaria (4.35 h) (18). However, it should be noted that drug exposure in the IBSM subjects (area under the concentration-time curve from 0 h to infinity [AUC_0–∞_], 1.95 μg · h/ml) was around 3-fold lower than was observed in the patients with naturally acquired malaria (AUC_0–∞_, 6.4 μg · h/ml) and similar to the exposure observed in healthy subjects dosed with 10 mg cipargamin in the first-in-human study (AUC_0–∞_, 1.86 μg · h/ml) (8). The dose-ranging phase 2a study also demonstrated increased clearance rates with higher single doses of cipargamin (parasite clearance half-life of 1.47 h following administration of the highest dose tested of 30 mg) (18). Furthermore, the ability of a multidose regimen of cipargamin (30 mg per day for 3 days) to rapidly clear parasitemia in both P. vivax and P. falciparum malaria patients was demonstrated in a separate phase 2 study (parasite clearance half-lives of 0.95 h for P. vivax malaria and 0.90 h for P. falciparum malaria) (10).

As anticipated from the dose selected in the current study, parasite regrowth occurred relatively rapidly in all subjects (3 to 8 days after cipargamin dosing), allowing the PK/PD relationship between parasitemia and cipargamin plasma concentration to be determined. However, the aforementioned limitation of the current study with respect to the enrollment of only a single-dose cohort is likely to have influenced the accuracy of these estimates. Since only one low dose was tested, it was not possible to achieve the higher exposure required to estimate the maximum killing effect (*E*_max_) and to investigate a dose-dependent killing effect. Thus, the MIC, MPC_90_, and minimum effective single dose reported here are likely to be overestimations. Indeed, the estimated MIC of 11.6 ng/ml was substantially higher than was estimated in patients with P. falciparum malaria (0.1 ng/ml) where different doses (10, 15, 20, and 30 mg) were tested (18). Where it has been possible to test multiple doses of experimental antimalarials in the IBSM model, the derived PK/PD relationship has been in alignment with that calculated from clinical studies involving patients with malaria presenting with significantly higher parasitemia (13, 21, 22).

Piperaquine monotherapy was successful in clearing asexual parasite regrowth (although second parasite regrowth did occur later in 3 subjects), allowing gametocytemia development to be investigated. Gametocytemia was observed in all subjects following piperaquine treatment, indicating that a single low dose of 10 mg cipargamin was not sufficient to prevent gametocyte development. Cipargamin was shown to be a potent inhibitor of both early- and late-stage gametocyte development *in vitro* (6). The inability of cipargamin to prevent gametocyte development in the current study could be due to insufficient exposure at the time of sexual commitment, or the fact that the sequestered early-stage gametocytes escape killing *in vivo* and the cipargamin blood concentration at the time the gametocytes reentered circulation was insufficient to kill mature gametocytes.

Transmission to mosquitoes was not observed for any subject when tested via direct membrane feeding assays. This is most likely due to the low density of gametocytes at the time of mosquito feeding (geometric mean of 439 female gametocytes/ml) since transmission is very unlikely to occur below 1,000 gametocytes/ml (23). It is possible that cipargamin treatment inhibited transmission by rendering the gametocytes infertile, but this cannot be concluded with such low gametocyte densities. Thus, the potent inhibition of oocyst development observed *in vitro* (6) warrants further clinical investigation into the transmission-blocking potential of cipargamin.

In conclusion, the *in vivo* antimalarial activity characterized in this study supports the further clinical development of cipargamin as a new treatment for P. falciparum malaria. The hepatic safety profile of cipargamin will be further evaluated in clinical development of the compound.

## MATERIALS AND METHODS

### Study design and participants.

This was a phase 1b, open-label, P. falciparum IBSM study conducted at Q-Pharm Pty. Ltd. (Brisbane, Australia). Healthy males and females (of non-child-bearing potential) of ages 18 to 55 years were eligible for inclusion in the study. Individuals were excluded if they had visited an area where malaria is endemic for a period greater than 4 weeks in the past 12 months or had received systemic therapy with a drug with potential antimalarial activity in the past 4 weeks. A complete list of the protocol-specified inclusion and exclusion criteria is included in Text S1 in the supplemental material. All subjects gave written informed consent before being included in the study. This study was approved by the QIMR Berghofer Medical Research Institute Human Research Ethics Committee and was registered at ClinicalTrials.gov with registration no. NCT02543086.

### Procedures.

A single cohort of eight subjects was inoculated intravenously with P. falciparum 3D7-infected human erythrocytes (approximately 1,800 viable parasites) as previously described (24). Parasite growth was monitored by collecting blood samples and performing quantitative PCR (qPCR) targeting the gene encoding P. falciparum 18S rRNA (25). Subjects received a single oral 10-mg dose of cipargamin (Novartis Pharmaceuticals, Switzerland) after an overnight fast when the protocol-specified threshold for commencement of treatment was reached (≥1,000 parasites/ml). The dose of cipargamin selected was predicted to be subtherapeutic based on preclinical modeling (7) and pharmacokinetic data obtained in previous clinical studies (8) and thus chosen to facilitate modeling to estimate the pharmacokinetic/pharmacodynamic relationship.

A single oral dose of 960 mg piperaquine (PCI Pharma Services, United Kingdom) was administered to subjects in response to parasite regrowth. The purpose of piperaquine treatment was to clear asexual parasites without clearing any gametocytes that may be present. This would allow the gametocytocidal activity of a second dose of cipargamin to be investigated, as well as the transmission of gametocytes to mosquitoes. Gametocytemia was monitored using quantitative reverse transcriptase PCR (qRT-PCR) targeting *pfs25* mRNA, a transcript preferentially expressed in mature female gametocytes (26). Transcripts per milliliter of blood were converted to female gametocytes per milliliter of blood using a standard curve (29). All participants were to receive compulsory rescue treatment with artemether-lumefantrine (Riamet; Novartis Pharmaceuticals Pty. Ltd., Macquarie Park, Australia), as well as a single 45-mg dose of primaquine (Primacin; BNM Group, Sydney, Australia) to clear gametocytes, 21 days after cipargamin dosing or earlier if a second round of parasite regrowth occurred following piperaquine treatment.

This study was planned to be conducted in two parts, with up to three-dose cohorts enrolled in part A following the procedures outlined above. Part B was planned to consist of single-dose cohort to specifically test the activity of a single dose of cipargamin against gametocytes. In this cohort, following inoculation, 480 mg piperaquine would be administered to clear asexual parasites when parasitemia reached a threshold of ≥5,000 parasites/ml, and cipargamin would subsequently be administered at peak gametocytemia (expected to occur approximately 15 days after piperaquine treatment). However, due to the occurrence of several serious adverse events (SAEs) following the initial dosing of cohort 1 in part A (see the “Safety” section in Results), the planned second dose of cipargamin was not administered, and the study was terminated without enrolling additional cohorts in part A or proceeding to part B.

### Safety assessment.

Safety assessments were performed throughout the study from screening and included adverse event reporting, physical examination, vital signs, clinical laboratory evaluation (hematology, biochemistry, and urinalysis), electrocardiography (ECG), and malaria clinical score recording. The malaria clinical score served as a clinical indication of the severity of the induced malaria infection; 14 signs and symptoms commonly associated with malaria were graded using a 3-point scale (0 = absent, 1 = mild, 2 = moderate, and 3 = severe), and the values were summed in order to generate an overall score (with a maximum possible score of 42). The severity of AEs was graded in accordance with the *Common Terminology Criteria for Adverse Events*, version 4.03 (https://evs.nci.nih.gov/ftp1/CTCAE/CTCAE_4.03/CTCAE_4.03_2010-06-14_QuickReference_5x7.pdf).

### Pharmacokinetic analysis.

Blood samples were collected at the following time points after dosing to determine the cipargamin plasma concentration: 1, 2, 3, 4, 8, 12, 16, 24, 36, 48, 72, 96, and 120 h. Samples were analyzed by liquid chromatography-tandem mass spectrometry as previously described (8). Noncompartmental PK analysis was performed using WinNonlin Pro version 6.4. Pharmacokinetic endpoints were the areas under the curve up to 24 h (AUC_0–24 h_), up to the last time point measure (AUC_0–last_), and extrapolated to infinity (AUC_0–∞_), the elimination half-life (*t*_1/2_), the peak plasma concentration (*C*_max_) and the time to *C*_max_ (*t*_max_), the apparent clearance (CL/F), and the apparent volume of distribution during the terminal phase (*V_z_*/F).

### Pharmacodynamic analysis.

To monitor total parasitemia, blood samples were collected for 18S rRNA qPCR before inoculation, on day 3, twice daily on days 4 to 6, pretreatment on day 7, and 4, 8, 12, 16, 24, 30, and 36 h posttreatment, twice daily from day 9 to day 11, and every 1 to 3 days until day 35. Blood samples for gametocytemia measurements were collected on day 11, every 1 to 3 days between day 15 and day 29, and on day 35. The pharmacodynamic variables of interest in this study were the parasite reduction ratio (PRR) and parasite clearance half-life, with the former expressed as the ratio of the parasite density decrease over a 48-h time period (expressed as the log_10_-transformed cohort-specific PRR over a 48-h time period [log_10_ PRR_48_]). Analyses were performed in R version 3.3.0. The PRR and parasite clearance half-life were estimated using the slope of the optimal fit for the log-linear relationship of the parasitemia decay as described previously (27).

### Pharmacokinetic/pharmacodynamic modeling.

Data preparation, exploration, model definition, model evaluation and simulations were performed in R (version 3.5.1) and the R package IQRtools (version 1.0.6; IntiQuan, Basel, Switzerland) within the software package MonolixSuite 2018R2 (Lixoft, Antony, France). The MIC and the minimum parasiticidal concentration that achieves 90% of maximum effect (MPC_90_) were derived from the PK/PD model. Simulations were performed to predict the minimum effective single dose, defined as the dose that clears 10^9^ parasites/ml. Further detail on PK/PD modeling methods is presented in Text S1.

### Transmission to mosquitoes.

The transmission of parasites from subjects to female Anopheles stephensi mosquitoes was measured using a direct membrane feeding assay (DMFA) as previously described (23). Briefly, ∼100 mosquitoes (3 to 7 days old) were allowed to feed on a water-jacketed membrane feeder filled with venous blood collected 20 days after cipargamin dosing into lithium heparin anticoagulant tubes. Seven to 9 days after blood feeding, mosquitoes were dissected to detect oocysts in midgut preparations using qPCR targeting the gene encoding 18S rRNA.

### Sample size.

The sample size of the current study (*n* = 8) was comparable to those of previous P. falciparum IBSM challenge studies and based on previously published experience was considered sufficient for obtaining statistically meaningful data on the effects of cipargamin on malaria parasite kinetics.

## Supplementary Material

Supplemental file 1
