# Supplementary material for: Defining the Antimalarial Activity of Cipargamin in Healthy Volunteers Experimentally Infected with Blood-Stage Plasmodium falciparum
Source: Antimicrob Agents Chemother. 2021 Jan 20;65(2):e01423-20. doi: 10.1128/AAC.01423-20 (PMC7849011; doi:10.1128/AAC.01423-20)
Supplement: Supplemental file 1 [file AAC.01423-20-s0001.pdf]

<u>**Supplementary methods and results**</u>

Defining the antimalarial activity of cipargamin in healthy volunteers experimentally infected with blood stage *Plasmodium falciparum*

James S. McCarthy, Azrin N. Abd-Rahman, Katharine A. Collins, Louise Marquart, Paul Griffin, Anne Kümmel, Aline Fuchs, Cornelis Winnips, Vishal Mishra, Katalin Csermak-Renner, J. Prakash Jain, Preetam Gandhi

Contents

Subject eligibility criteria 2

Safety results 6

Table S1. Incidence of adverse events by system organ class and preferred term 6

Table S2. Malaria clinical score recorded for each subject at the time of cipargamin dosing and peak score recorded during the study 7

Figure S1. Liver function test results over the entire study for the three subjects who displayed abnormalities classified as serious 7

Individual subject parasite clearance rates following cipargamin treatment 8

Table S3. Individual $\log_{10}PRR_{48}$ and parasite clearance half-life 8

Pharmacokinetic/Pharmacodynamic modelling methods and results 9

Table S4. Parameter estimates of the final population pharmacokinetic model 11

Figure S2. Visual predictive check of the final population pharmacokinetic model 12

Table S5. Parameter estimates of the final population pharmacokinetic-pharmacodynamic model 13

Table S6. Efficacy parameters following administration of 10 mg cipargamin 13

Figure S3. Visual predictive check of the final population pharmacokinetic-pharmacodynamic model 14

Figure S4. Simulated total PRR following administration of different cipargamin single doses 15

References 16

Subject eligibility criteria

Inclusion criteria

Subjects eligible for inclusion in this study were required to fulfil all of the following criteria:

1. Written informed consent had to be obtained before any assessment was performed.

Demography

2. Male and females participants between 18 and 55 years of age, inclusive who did not live alone (from Day -7 until at least the end of the antimalarial drug treatment) and were contactable and available for the duration of the trial (maximum of 4 months). Female participants between 18 and 55 years of age were also allowed as long as they were of non-child bearing potential. Women were considered post-menopausal and not of child bearing potential if they had 12 months of natural (spontaneous) amenorrhea with an appropriate clinical profile (e.g. age appropriate, history of vasomotor symptoms) or had surgical bilateral oophorectomy (with or without hysterectomy) or tubal ligation at least six weeks prior to participation on the study . In the case of oophorectomy alone, only when the reproductive status of the woman had been confirmed by follow up hormone level assessment was she considered not of child bearing potential. A pregnancy test was performed on all female subjects, regardless of reported reproductive status.

3. Body weight, minimum 50.0 kg, body mass index (BMI) between 18.0 and 32.0 kg/m^2, inclusive.

Health status

4. Certified as healthy by a comprehensive clinical assessment (detailed medical history and complete physical examination (PE)).

- Normal vital signs after 5 minutes resting in supine position:
- 90 mmHg < systolic blood pressure (SBP) <140 mmHg,
- 50 mmHg < diastolic blood pressure (DBP) <90 mmHg,
- 40 bpm< heart rate (HR) <100 bpm.

5. Normal standard 12-lead electrocardiogram (ECG) after 5 minutes resting in supine position; QT interval with Fridericia correction (QTc) ≤450 ms (females) and <430 ms (males) with absence of second or third degree atrioventricular block or abnormal T wave morphology.

6. Laboratory parameters within the normal range.

In the case where a laboratory range was not specified by the protocol, but was outside the reference range for the center at Screening and/or initial Baseline, a decision regarding whether the result were of clinical significance or not was made by the Investigator and was based, in part, upon the nature and degree of the observed abnormality.

7. Participants agreed to use a double barrier method of contraception including condom plus diaphragm or condom plus intrauterine device or condom plus stable oral/transdermal/injectable hormonal contraceptive by female partner for at least 14 days prior to the time of the first dose of study drug through 95 days (i.e. $5 \times T_{1/2}$ + 90 days for a

full spermatogenesis cycle) after the last dose of study drug. Abstinent participants had to agree starting a double barrier method if they started sexual relationships during the study and up to 95 days after the last dose of study drug.

Exclusion criteria

Subjects fulfilling any of the following criteria were not eligible for inclusion in this study.

Medical history and clinical status

1. Any history of malaria or participation to a previous malaria challenge study.

2. Spent more than four weeks in a malaria-endemic country during the past 12 months or planned travel to a malaria-endemic area during the course of the study.

3. Had evidence of increased cardiovascular disease risk (defined as >10%, 5 year risk). Risk factors include sex, age, systolic blood pressure (mm/Hg), smoking status, BMI (kg/m^2) and diabetes mellitus.

4. History of splenectomy.

5. Presence or history of drug hypersensitivity, or allergic disease diagnosed and treated by a physician or history of a severe allergic reaction, anaphylaxis or convulsions following any vaccination or infusion.

6. Presence of current or suspected serious chronic diseases such as cardiac or autoimmune disease (human immunodeficiency virus (HIV) or other immunodeficiencies), insulindependent and non-insulin dependent diabetes, progressive neurological disease, severe malnutrition, acute or progressive hepatic disease, acute or progressive renal disease, psoriasis, rheumatoid arthritis, asthma, epilepsy or obsessive compulsive disorder.

7. History of malignancy of any organ system (other than localized basal cell carcinoma of the skin or in-situ cervical cancer), treated or untreated, within the past 5 years, regardless of whether there was evidence of local recurrence or metastases.

8. History of photosensitivity.

9. Participants with history of schizophrenia, bi-polar disease, or other severe (disabling) chronic psychiatric diagnosis, including depression or receiving psychiatric drugs or who had been hospitalized within the past 5 years prior to enrollment for psychiatric illness, history of suicide attempt or confinement for danger to self or others.

10. Frequent headaches and/or migraine, recurrent nausea, and/or vomiting (more than twice a month).

11. Presence of acute infectious disease or fever (e.g., sub-lingual temperature ≥ 38.5°C) within the five days prior to inoculation with malaria parasites.

12. Evidence of acute illness within the four weeks before trial prior to Screening that the Investigator deemed may compromise subject safety.

13. Significant inter-current disease of any type, in particular liver, renal, cardiac, pulmonary, neurologic, rheumatologic, or autoimmune disease by history, physical examination, and/or laboratory studies including urinalysis.

14. Participant had a clinically significant disease or any condition or disease that might affect drug absorption, distribution or excretion, e.g. gastrectomy, diarrhoea.

15. Participation in any investigational product study within the 12 weeks preceding the study.

16. Participation in any research study involving blood sampling (more than 450 mL/ unit of blood), or blood donation to Red Cross (or other) blood bank during the 8 weeks preceding the reference drug dose in the study.

17. Participant unwilling to defer blood donations to the Australian Red Cross Blood Service (ARCBS) for 6 months.

18. Medical requirement for intravenous immunoglobulin or blood transfusions.

19. Participant who had ever received a blood transfusion.

20. Symptomatic postural hypotension at Screening, irrespective of the decrease in blood pressure, or asymptomatic postural hypotension defined as a decrease in systolic blood pressure ≥20 mmHg within 2-3 minutes when changing from supine to standing position.

21. History or presence of alcohol abuse (alcohol consumption more than 40 g per day for the preceding 3 months) or drug habituation, or any prior intravenous usage of an illicit substance.

22. Smoking more than 5 cigarettes or equivalent per day and unable to stop smoking during the study.

23. Ingestion of any poppy seeds within the 24 hours prior to the Screening blood test (participants were to be advised by phone not to consume any poppy seeds in this time period).

24. Excessive consumption of beverages containing xanthine bases, including red bull, chocolate, etc., (e.g, more than 400 mg of caffeine per day or more than 4 cups or glasses per day).

<u>Interfering substance</u>

25. Any vaccination within the last 28 days.

26. Any corticosteroids, anti-inflammatory drugs, immunomodulators or anticoagulants. Any participant currently receiving or having received in the previous 3 months immunosuppressive therapy, including systemic steroids including adrenocorticotrophic hormone (ACTH) or inhaled steroids in dosages are associated with hypothalamicpituitary-adrenal axis suppression such as 1 mg/kg/day of prednisone or its equivalent or chronic use of inhaled high potency corticosteroids (budesonide 800 μg per day or fluticasone 750 μg).

27. Any recent (<1 month) or current systemic therapy with an antibiotic or drug with potential antimalarial activity (including chloroquine, PQP (BYQ709), tetracycline, azithromycin, clindamycin, hydroxychloroquine).

28. Medicinal products known to prolong the QTc interval.

29. Any of the substances listed in Table 9-2 of the study protocol. Prohibited medications due to potential drug-drug or PD interactions taken within 14 days prior to the study

General conditions

30. Any participant who, in the judgment of the Investigator, was likely to be non-compliant during the study, or unable to cooperate because of a language problem or poor mental development.

31. Any participant in the exclusion period of a previous study according to applicable regulations.

32. Any participant who was the Investigator or any sub-Investigator, research assistant, pharmacist, study coordinator, or other staff thereof, directly involved in conducting the study.

33. Any participant without a good peripheral venous access.

Biological status

34. Positive result on any of the following tests: hepatitis B surface (HBs Ag) antigen, antihepatitis B core antibodies (anti-HBc Ab), anti-hepatitis C virus (anti-HCV) antibodies, anti-human immunodeficiency virus 1 and 2 antibodies (anti-HIV1 and anti HIV2 Ab).

35. Any drug as listed in Appendix 6 of the study protocol in the urine drug screen unless there was an explanation acceptable to the medical Investigator (e.g., the participant had stated in advance that they consumed a prescription or over the counter product which contained the detected drug) and/or the participant had a negative urine drug screen on retest by the pathology laboratory.

Specific to the study

36. Family history of sudden death or of congenital prolongation of the QTc interval or known congenital prolongation of the QTc-interval or any clinical condition known to prolong the QTc interval. History of symptomatic cardiac arrhythmias or with clinically relevant bradycardia. Electrolyte disturbances, particularly hypokalemia, hypocalcaemia or hypomagnesaemia.

37. Electrocardiogram abnormalities in the standard 12-lead ECG (at Screening) which in the opinion of the Investigator was clinically relevant or would interfere with the ECG analysis.

38. Known hypersensitivity (active substance and/or excipients) to cipargamin, PQP, artemether, lumefantrine or primaquine.

39. Known severe reaction to mosquito bites other than local itching and redness.

40. Unwillingness to abstain from consumption of certain citrus fruits (grapefruit, Seville orange, etc.) or their juices, as well as quinine containing foods/beverages such as tonic water, lemon bitter, from inoculation to the end of the malaria treatment.

41. Any history or presence of lactose intolerance.

42. Unwillingness to use commonly recommended sun-protection methods (e.g. sunscreen, hat, protective clothing) after using cipargamin.

Safety results

Table S1. Incidence of adverse events by system organ class and preferred term

**System Organ Class**<br>Preferred Term	**10 mg cipargamin [N=8]**<br>**n (%)**
Subjects with at least one adverse event	8 (100)
**Cardiac disorders**	**1 (12.5)**
Tachycardia	1 (12.5)
**Eye disorders**	**1 (12.5)**
Photophobia	1 (12.5)
**Gastrointestinal disorders**	**5 (62.5)**
Abdominal discomfort	4 (50.0)
Diarrhoea	2 (25.0)
Nausea	1 (12.5)
**General disorders and administration site conditions**	**6 (75.0)**
Chills	3 (37.5)
Malaise	4 (50.0)
Non-cardiac chest pain	1 (12.5)
Pyrexia	6 (75.0)
**Hepatobiliary disorders**	**1 (12.5)**
Drug-induced liver injury	1 (12.5)
Liver tenderness	1 (12.5)
**Investigations**	**3 (37.5)**
Alanine aminotransferase increased	2 (25.0)
Aspartate aminotransferase increased	1 (12.5)
Gamma-glutamyltransferase increased	2 (25.0)
Liver function test abnormal	1 (12.5)
**Metabolism and nutrition disorders**	**3 (37.5)**
Decreased appetite	3 (37.5)
**Musculoskeletal and connective tissue disorders**	**5 (62.5)**
Arthralgia	2 (25.0)
Myalgia	5 (62.5)
Neck pain	1 (12.5)
**Nervous system disorders**	**6 (75.0)**
Head discomfort	1 (12.5)
Headache	5 (62.5)
Lethargy	4 (50.0)
**Respiratory, thoracic and mediastinal disorders**	**3 (37.5)**
Haemoptysis	1 (12.5)
Rales	1 (12.5)
Rhinorrhoea	1 (12.5)
**Skin and subcutaneous tissue disorders**	**4 (50.0)**
Hyperhidrosis	3 (37.5)
Rash	1 (12.5)
Urticaria	1 (12.5)

A subject with multiple occurrences of an adverse event is counted only once in the AE category.

Table S2. Malaria clinical score recorded for each subject at the time of cipargamin dosing and peak score recorded during the study

Subject	Clinical score at time of cipargamin dosing	Peak clinical score during study	Time of peak clinical score (days post-dosing)
1	0	0	Not applicable
2	1	6	7
3	0	7	7
4	0	3	2
5	0	4	5
6	0	6	8
7	0	0	Not applicable
8	0	13	7

The malaria clinical score served as a clinical indication of the severity of the induced malaria infection; 14 signs and symptoms commonly associated with malaria were graded using a 3-point scale (0=absent, 1=mild, 2=moderate, 3=severe) and the values were summed in order to generate an overall score (maximum possible score is 42).

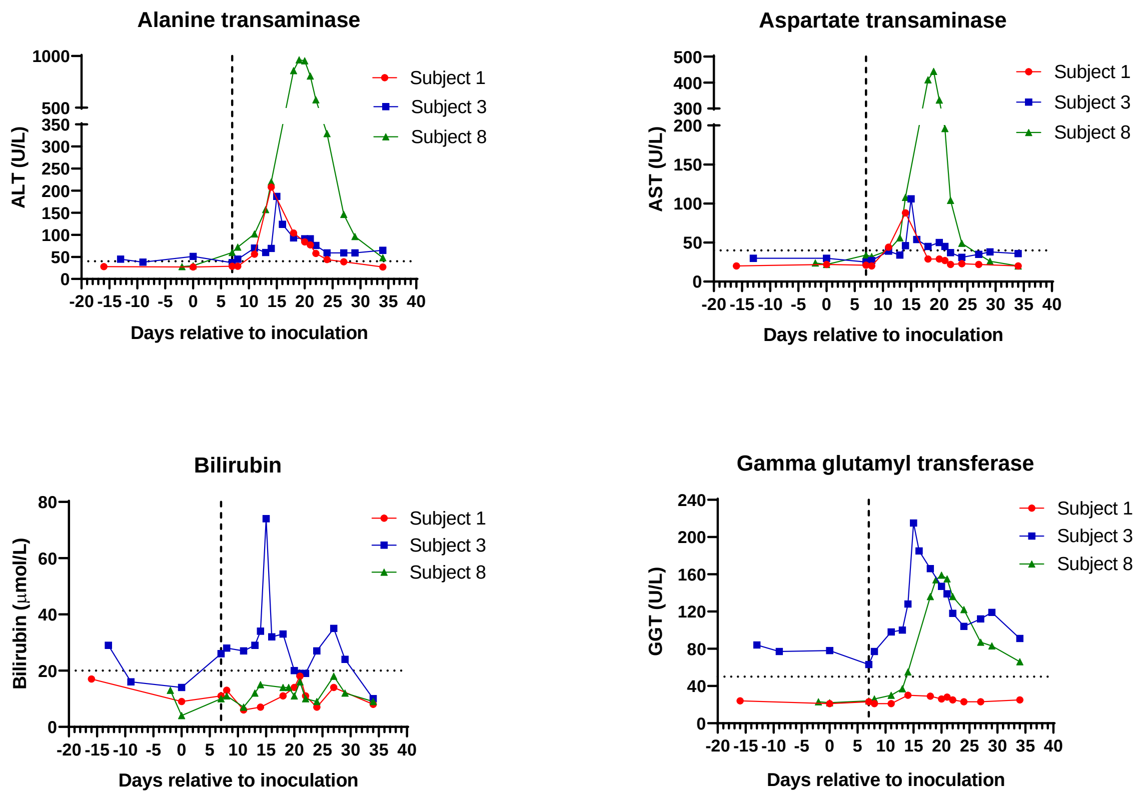

Figure S1. Liver function test results over the entire study for the three subjects who displayed abnormalities classified as serious

The vertical dashed lines indicate the day of dosing with 10 mg cipargamin. The horizontal dotted lines indicate the upper limit of normal for each LFT parameter.

Individual subject parasite clearance rates following cipargamin treatment

Table S3. Individual $\log_{10}PRR_{48}$ and parasite clearance half-life

**Subject**	**$Log_{10}PRR_{48}$ (95%CI)**	**Parasite Clearance Half-Life (h) (95%CI)**	**P-value**
1	4.22 (3.15, 5.29)	3.42 (2.73, 4.59)	0.0002443
2	5.84 (4.79, 6.90)	2.47 (2.10, 3.02)	0.0000357
3	4.09 (3.45, 4.73)	3.53 (3.05, 4.19)	0.0000161
4	2.18 (1.62, 2.73)	6.64 (5.30, 8.90)	0.0000161
5	4.23 (3.48, 4.98)	3.42 (2.90, 4.15)	0.0000323
6	1.28 (0.85, 1.71)	11.39 (8.47, 17.37)	0.000201
7	4.12 (3.22, 5.03)	3.51 (2.87, 4.49)	0.0001104
8	4.63 (4.34, 4.92)	3.12 (2.93, 3.33)	7.4494E-8

$Log_{10}PRR_{48}$: logarithm to the base 10 of the parasite reduction ratio standardized over a 48 hour period after treatment; CI: confidence interval.

Pharmacokinetic/Pharmacodynamic modelling methods and results

Objectives

The objectives of the PK/PD modelling analysis were:

1. To develop a population PK model of cipargamin in healthy subjects inoculated with blood stage *P. falciparum*.
2. To characterize the PK/PD relationship between cipargamin pharmacokinetics and parasite clearance in health subjects inoculated with blood stage *P. falciparum*.
3. To determine the minimum inhibitory concentration (MIC) of cipargamin.
4. To establish the minimum effective cipargamin single dose required to kill 10^9 parasites.

Methods

Software

Data preparation, exploration, model definition, model evaluation and simulations were performed in R (version 3.5.1) and the R package IQRtools (version 1.0.6, IntiQuan GmbH, Basel, Switzerland) within the software package MonolixSuite2018R2 (Lixoft, Antony, France) which implements stochastic approximation expectation-maximisation (SAEM) algorithm for parameter estimation.

Population PK modelling

One-, two- and three-compartment with different absorption and elimination models were tested. Zero- and first-order absorption models with or without lag time were examined. Data below the lower limit of quantitation (LLOQ; 0.001 µg/mL) were treated using a left-censoring method. Between subject variability (BSV) of the PK parameters was assumed to be normally distributed on log-scale. The random effects across the parameters within an individual were modelled according to a multivariate normal distribution (MVN). BSV was tested on all PK parameters, but it was fixed to zero if their inclusion did not adequately supported by the data. The residual unexplained variability (RUV) was tested using a proportional or combined error models.

Population PK/PD modelling

A sequential PK/PD modelling approach was used in which the empirical Bayes estimates of individual PK parameters were used as regression parameters for the PK/PD model. The observations below the limit of detection (LOD; 10 parasites/mL) were modelled using a left-censoring method. In this analysis, the changes in parasitemia over time in the presence of antimalarial drug are assumed to be a result of the difference between parasite growth rate (k_{grow}) and the rate at which parasites are killed by the drug (k_{kill}) with a baseline parasitemia (PL_{base}) at time t_0 (time of inoculation). Different structural PK/PD models were tested to assess the relationship of cipargamin plasma concentration on the parasite killing rate. The E_{max} model assumed a direct effect of cipargamin plasma concentrations on parasite killing. The turnover model assumed an indirect effect of cipargamin plasma concentrations on parasite killing due to lag in the PD processes (i.e. the kinetics of drug-receptor binding). The effect compartment model assumed an indirect effect of cipargamin plasma concentration on parasite killing due to biophase equilibration. This model can be interpreted as the delay for the drug to reach the site of action in which the cipargamin concentrations in the effect

compartment drives the drug effect. The parasite clearance model assumed that the observed parasites were a mixture of living and dead parasites and that the cipargamin plasma concentrations had a direct effect on the killing of the parasites.

PD parameters were modelled assuming a log-normal distribution with exception of ln-transformed baseline parasite values (parasitemia at time of inoculation) where a normal distribution was assumed. BSV on k_{grow} was estimated and BSV on other PD parameters were fixed to 0.2. RUV was modelled as an additive error on the logarithmically transformed observations, which corresponded to an exponential error on an arithmetic scale. During the base model building, the PD parameters were estimated based on fixed Hill coefficients and these coefficients were fixed to 1, 2, 3, 5, 7 and 10. For each coefficient, four estimations were made for the PD parameters with randomised initial values.

Calculation of key efficacy parameters

The minimum inhibitory concentration (MIC), the minimal parasiticidal concentration at 90% maximum effect (MPC_{90}) and the parasite reduction rate within 48 h post-dose (PRR_{48}) were derived from the PD models. MIC was defined as the lowest drug concentration which inhibited parasite growth. MPC_{90} was defined as the lowest concentration needed at which the parasite killing rate was equal to 90% of its maximum. $PRR_{48\ max}$ was defined as the maximum parasite reduction ratio achieved within 48 h. The $PRR_{48\ max}$ was determined by assuming a concentration well above MPC_{90} for this time span. $PRR_{48\ actual}$ was defined as the parasite reduction ratio achieved within the first 48 h after dose administration. The $PRR_{48\ actual}$ was determined by simulation as the difference of predicted parasite levels between the time of dose administration and 48 h later. Both PRR_{48} were expressed in log_{10}-transformed scale.

Model evaluation

The model goodness of fit was assessed using the diagnostic plots of observations versus population and individual predictions, plots of population weighted residuals (PWRES), individual weighted residuals (IWRES) and normalised prediction distribution errors (NPDE) versus time and population predictions. Parameter estimate shrinkage (eta shrinkage) was computed to evaluate the reliability of empirical Bayes estimates and individual prediction values in the assessment of goodness of fit plots. In addition, successful convergence of the minimisation routine, reasonable physiological plausibility and precision of parameter estimates were also taken into account during the model development. The predictive performance of the final model was evaluated using the visual predictive check (VPC) in which 1000 observations at each time points were simulated from the final model. The 5th, 50th and 95th percentiles their respective simulated 95% CI were plotted against time.

Simulation for dose prediction to clear 10^9 parasites/mL

Once the population PK/PD model was developed in healthy volunteers inoculated with blood stage *P. falciparum* malaria, simulations were undertaken in patients with *P. falciparum* for a range of single oral doses of cipargamin. Simulated cipargamin doses were 10, 50, 100, 120, 300, 400, 500, 600, 700, 800, 900, 1000, 1200, 1500, 2000, 2500, 3000, 5000, 7500 and 10000 mg. The PK model and PK/PD relationship were assumed to be the same in patients as in volunteers. The parasites were assumed to grow exponentially in patients at growth rate of 0.048 h^{-1}, which is equivalent to multiplication rate of

approximately 10 fold per asexual cycle of 48 h. The baseline parasitemia was assumed to be log-normally distributed around the median value of 10^7 parasites/mL. A total of 500 patients were simulated for each dose in which population parameters were sampled from the uncertainty distribution. The cipargamin plasma concentration-time and parasitemia-time profiles were simulated up to day 28. The minimum effective dose was defined as the minimum dose that resulted in parasite reduction ratio over the simulated time of 28 days (PRR_{tot}) of at least 10^9.

Results

<u>Population PK modelling</u>

A two-compartment disposition model with first-order absorption and elimination and a proportional error adequately described the cipargamin plasma concentration-time profiles. A two compartment model has been reported in *P. falciparum* malaria patients receiving cipargamin (1). Apparent clearance (CL/F) and apparent volume of distribution for central compartment (V_c/F) were correlated. BSV on absorption rate constant (k_a), apparent volume of distribution for peripheral compartment 1 (V_{p1}/F) and apparent inter-compartmental clearance between central compartment and peripheral compartment 1 (Q_1/F) were estimated with unacceptably low precision [relative standard error (RSE) >60%] and therefore was fixed to 0.1. The relative bioavailability cannot be estimated and was assumed to be 100% since cipargamin was only administered orally. No covariates were tested due to small number of subjects. The PK parameter estimates of the final population PK model are summarised in Table S4. The visual predictive check (VPC plot; Figure S2) demonstrated that the observed cipargamin plasma concentration-time profile at the 5th, 50th and 95th percentiles fell within the simulated predictions, indicating the model adequacy in characterising the observed data.

Table S4. Parameter estimates of the final population pharmacokinetic model

Parameters	Final population PK model		
	Estimate[a]	BSV[b] (%)	Shrinkage[c] (%)
F	1 (fixed)	0 (fixed)	-
k_a (/h)	0.919 (4.6)	0.1 (fixed)	-
CL/F (L/h)	5.5 (10.7)	0.325 (23.3)	-6.2
V_c/F (L)	64.4 (8.9)	0.227 (29.3)	-2.2
Q_1/F (L/h)	12.9 (9.0)	0.1 (fixed)	-
V_{p1}/F (L)	107 (4.8)	0.1 (fixed)	-
$\rho_{CL/F,Vc/F}$	0.763 (23.3)	-	-
ε_{prop}	0.111 (8.3)	-	-

PK = pharmacokinetic, CL/F = apparent clearance, V_c/F = apparent volume of distribution for central compartment, Q_1/F = apparent inter-compartmental clearance between central and peripheral 1 compartments, V_{p1}/F = apparent volume of distribution for peripheral 1 compartment, F = relative bioavailability of first-order absorption, ρ = correlation coefficient, ε_{prop} = proportional error model.

[a] Estimate, with between brackets the RSE (relative standard error %).

[b] Standard deviation of the between subject variability in the parameter, with between brackets the RSE.

[c] Shrinkage for between subject variability.

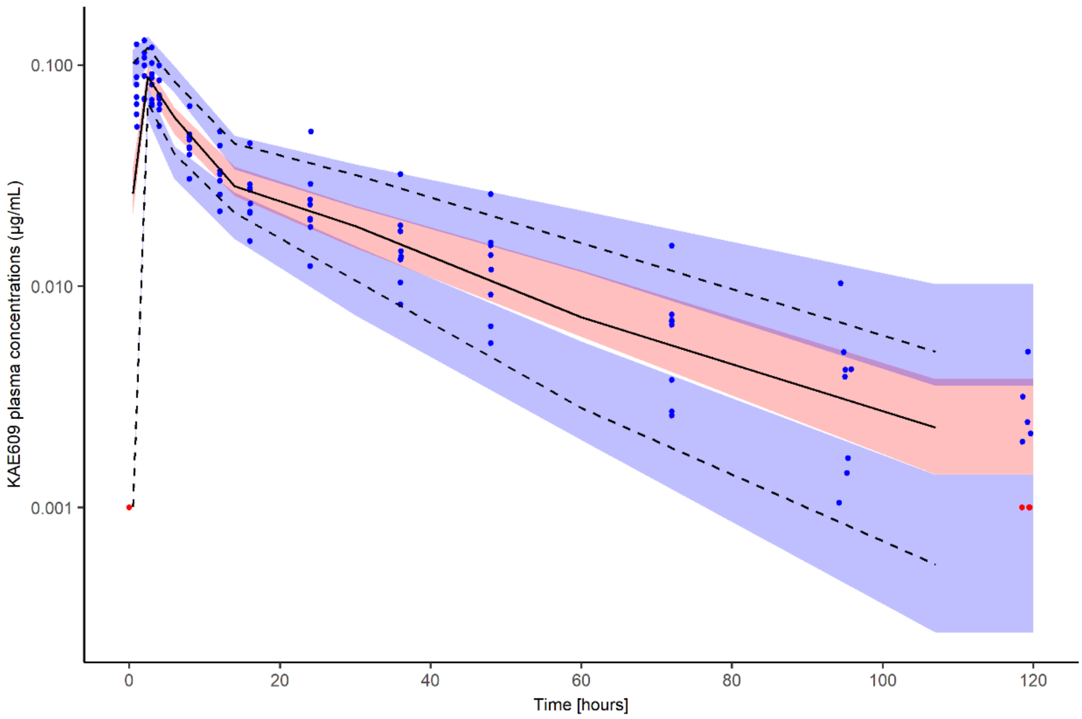

Figure S2. Visual predictive check of the final population pharmacokinetic model

The solid blue circles represent the measured cipargamin (KAE609) plasma concentrations, the solid red circles represent the simulated below the lower limit of quantification cipargamin plasma concentrations, the black solid lines represent the 50th percentile for the measured cipargamin plasma concentrations, the black dashed lines represent the 5th and 95th percentiles for the measured cipargamin plasma concentrations, the blue shaded areas represent the confidence intervals for 5th and 95th percentiles and the pink shaded area the 95% confidence interval for the 50th percentile derived from 1000 stochastic profiles simulated from the final population pharmacokinetic model.

Population PK/PD modelling

The relationship between cipargamin plasma concentration and parasite killing was tested using different PK/PD models. Table S5 shows the comparison of the parameter estimates of the final PK/PD model selected (E_{max} model). Figure S3 shows the VPC plot for the final PK/PD model. Overall, the 5th, 50th and 95th percentiles of the observed data were within the 95% CI of the simulated data. Efficacy results following administration of 10 mg cipargamin are presented in Table S6.

Table S5. Parameter estimates of the final population pharmacokinetic-pharmacodynamic model

Parameter	E_{max} model	
BIC	499	
	**Estimate**	**RSE (%)**
Pharmacodynamic parameter estimates		
PL_{base} (Ln parasite/mL)	-3.75	15
k_{grow} (/h)	0.0714	5.7
E_{max} (/h)	0.23	9.7
EC_{50} (µg/mL)	0.0151	9.8
Hill coefficient	5 (fixed)	-
k_{in}(/h)		
$CL_{parasite}$ (/h)		
k_e (/h)		
Between subject variability		
$\omega_{PL_{base}}$	0.2 (fixed)	-
$\omega_{k_{grow}}$	0.0242	100
$\omega_{E_{max}}$	0.2 (fixed)	-
$\omega_{EC_{50}}$	0.2 (fixed)	-
$\omega_{k_{in}}$		
$\omega_{CL_{parasite}}$		
ω_{k_e}		
Residual unexplained variability		
ε_{add}	1.24	5.4

BIC = Bayesian information criterion, RSE = relative standard error, PL_{base} = parasitemia at baseline (time of inoculation), k_{grow} = parasite growth rate constant, E_{max} = maximum parasite killing rate, EC_{50} = concentration of drug that produce half of the E_{max}, k_{in} = turnover rate constant, $CL_{parasite}$ = parasite clearance rate, k_e = transfer rate constant to and from the effect compartment, $\omega_{parameter}$= between subject variability in the parameter, ε_{add} = additive residual error on log scale (equivalent to proportional error model on ordinary scale).

Table S6. Efficacy parameters following administration of 10 mg cipargamin

Parameters	Estimates[a]
MIC (ng/mL)	11.6 (9.9 – 13.3)
MPC_{90} (ng/mL)	23.5 (19.9 – 27.4)
$PRR_{48\ actual}$ (log_{10} unit)	2.59 (1.89 – 3.32)
Time above MIC (days)	1.96 (1.59 –2.44)
Time above MPC_{90} (days)	0.89 (0.63–1.26)

MIC = minimum inhibitory concentration, MPC_{90} = minimum parasiticidal concentration at 90% of maximum effect, $PRR_{48\ actual}$ = parasite reduction ratio over 48 h. [a]Estimates are in median (95% CI).

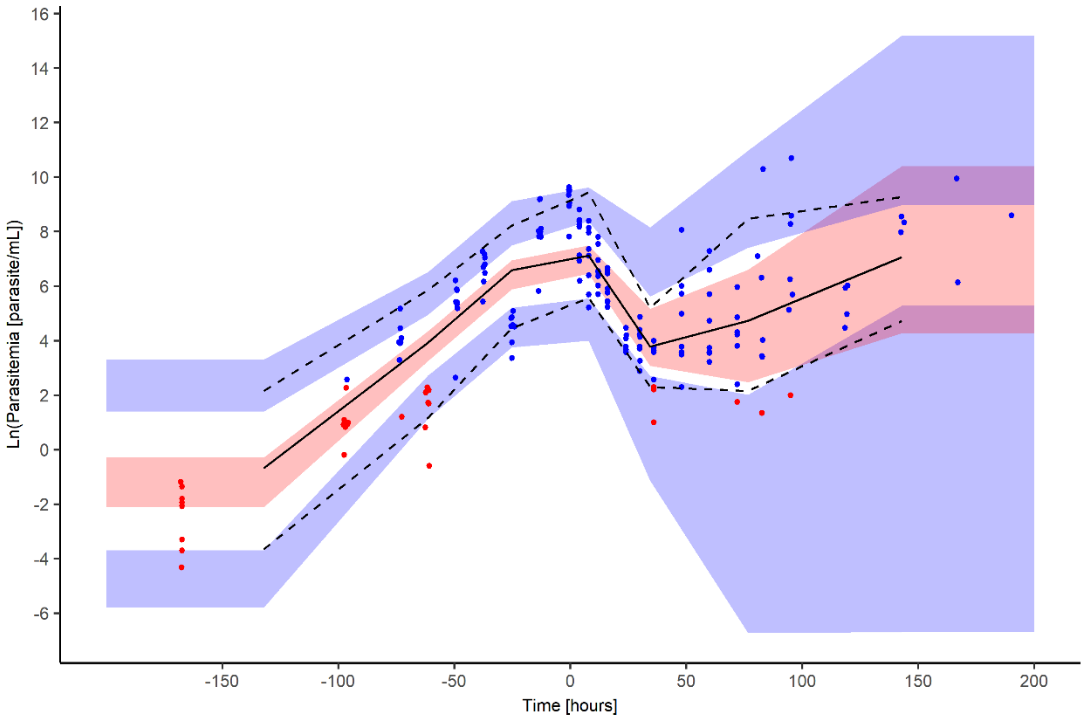

Figure S3. Visual predictive check of the final population pharmacokinetic-pharmacodynamic model

The solid blue circles represent the measured *P. falciparum* parasitemia, the solid red circles represent the simulated below the lower limit of quantification *P. falciparum* parasitemia, the black solid lines represent the 50th percentile for the measured *P. falciparum* parasitemia, the black dashed lines represent the 5th and 95th percentiles for the measured *P. falciparum* parasitemia, the blue shaded areas represent the confidence intervals for 5th and 95th percentiles and the pink shaded area the 95% confidence interval for the 50th percentile derived from 1000 stochastic profiles simulated from the final population pharmacokinetic-pharmacodynamic model.

Simulation for dose prediction to clear 10^9 parasites/mL

Figure S4 illustrates how PRR_{total} changes with dose for a trial of 500 patients. The minimum dose that clears at least 10^9 parasites/mL is 95 mg (95% CI: 50-270).

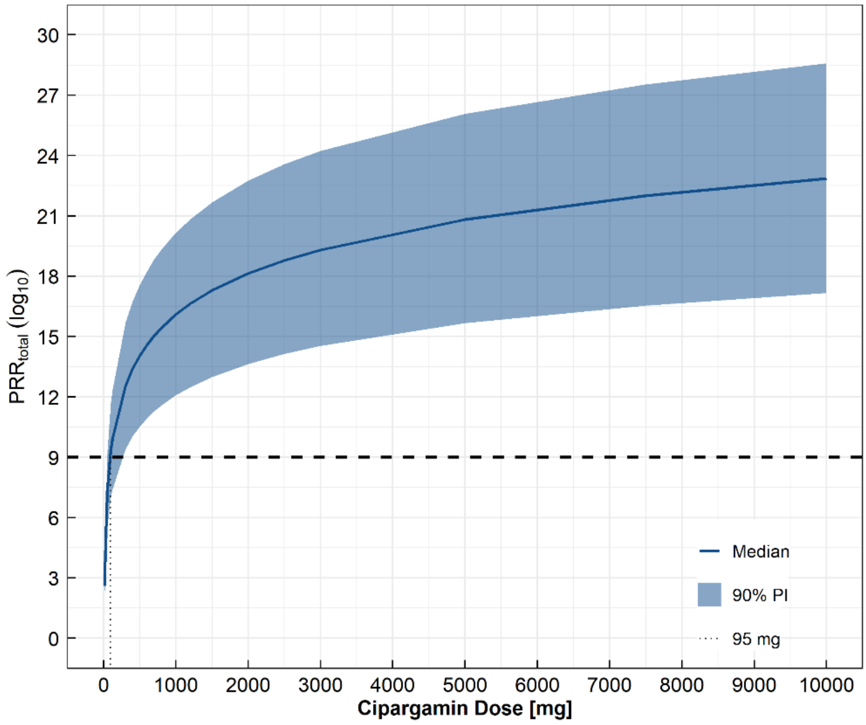

Figure S4. Simulated total PRR following administration of different cipargamin single doses

The blue solid line represents the median PRR_{total}, the blue shaded area represents the 90% prediction interval, the black dashed line represents 10^9 parasite kill. Simulations were performed in 500 hypothetical patients, PRR_{total} = total parasite reduction ratio.

References

1. Hien TT, White NJ, Thuy-Nhien NT, Hoa NT, Thuan PD, Tarning J, Nosten F, Magnusson B, Jain JP, Hamed K. 2017. Estimation of the in vivo MIC of cipargamin in uncomplicated *Plasmodium falciparum* malaria. Antimicrob Agents Chemother 61:e01940-16.
